# Intestinal mucosal injury induced by obstructive jaundice is associated with activation of TLR4/TRAF6/NF-κB pathways

**DOI:** 10.1371/journal.pone.0223651

**Published:** 2019-10-31

**Authors:** Xiaopeng Tian, Huimin Zhao, Zixuan Zhang, Zengcai Guo, Wen Li

**Affiliations:** 1 Medical School of Chinese PLA, Beijing, China; 2 Department of Gastroenterology, Xingtai People’s Hospital, Xingtai, Hebei, China; 3 Department of Gastroenterology and Hepatology, the First Medical Center of Chinese PLA General Hospital, Beijing, China; Texas A&M University, UNITED STATES

## Abstract

**Objectives:**

To investigate the role of TLR4/TRAF6/NF-κB pathways in intestinal mucosal injury induced by obstructive jaundice (OJ).

**Methods:**

A total of 100 male C57BL/6J mice were randomly assigned to two groups: (I) sham operation (SH); (II) OJ. The mice were sacrificed before operation and on the 1^st^, 3^rd^, 5^th^ and 7^th^ day after operation. The blood and terminal ileum were simultaneously collected under the aseptic condition for further detection.

**Results:**

In the SH group, TLR4 protein and mRNA rarely expressed in the intestinal mucosa of the mice and there were no significant differences at different time points (*p*>0.05). By contrast, in the OJ group TLR4 protein (0.12±0.06, 0.16±0.08, 0.27±0.10, 0.35±0.12 and 0.41±0.13, respectively) and mRNA (0.49±0.19, 0.62±0.23, 0.98±0.32, 1.42±0.41 and 1.72±0.49, respectively) increased gradually with the extension of time (*p*<0.05). Also in the OJ group, the levels of DAO and endotoxin in plasma as well as the expressions of NF-κB and caspase-3 increased gradually with the extension of time, showing positive correlation with the expression of TLR4 (*p*<0.05).

**Conclusions:**

The expression of TLR4 was significantly up-regulated in the distal ileum of mice with OJ. Activation of the TLR4/TRAF6/NF-κB pathways was involved in the occurrence and development of intestinal mucosal injury and endotoxemia in mice with OJ.

## Introduction

Obstructive jaundice (OJ) is a syndrome which is caused by the obstruction of biliary tract leading to impaired liver function, and then inducing a series of pathological and physiological changes in various systems of the whole body. As OJ progresses, it may occur multiple bacterial infections frequently, which may result in hepatorenal syndrome, coagulation dysfunction, impaired intestinal mucosal barrier, MODS and even death in severe cases [1]. Surgery is the only effective method to address the obstruction of biliary tract at present. Under normal physiological conditions, the body can effectively prevent intestinal bacteria and endotoxin from entering tissues and organs other than the intestine through intestinal mucosal barrier. However, in the case of the obstruction of biliary tract, the intestinal environment changes greatly, exhibiting damaged intestinal mucosal barrier and increased intestinal permeability, resulting in bacterial translocation and endotoxemia [2–4].

Toll-like receptors (TLRs) is a class of transmembrane receptors that widely exists in mammalian cells. TLRs can recognize a number of conservative pathogen-associated molecular patterns (PAMPs) of pathogenic microorganism, initiate signal transduction, eventually stimulate the activation of nuclear factor kappa B (NF-κB) and induce inflammatory mediator expression. TLRs may thus play a key role in innate immune system [5]. Among them, TLR4 can identify the lipopolysaccharide (LPS) of Gram-negative bacteria, which is one of the principal pathways for host identification of Gram-negative bacteria infection [6]. In recent years, the role of TLR4 has become a hot topic again in various pathogenesis of intestinal diseases, such as inflammatory bowel disease (IBD) [7–9]. However, it remains unclear how TLR4 and its downstream signaling pathways play a role in the pathogenesis of OJ. The aim of this study was to investigate the expression changes of intestinal TLR4 and its downstream signaling pathways in the process of experimental OJ, and to analyze their roles in intestinal mucosal injury.

## Materials and methods

### Animal models

C57BL/6J mice (n = 100; adult male; 20**~**24 g) were used in this study. All mice were purchased from the Laboratory Animal Center of Academy of Military Medical Sciences and housed in the Experimental Animal Service Center of the Xingtai People’s Hospital. The study was approved by the Animal Research Ethics Committee of the Xingtai People’s Hospital. The mice were fed with a standard rat food before and after surgery. They were randomly placed in two groups: (I) sham operation (SH, n = 50); (II) OJ (n = 50); The mice in the OJ group had the common bile duct ligated above the pancreas, and then cut and sutured to establish the model of bile duct ligation (BDL) on day 0. SH was performed with loose ligation of the common bile duct. The mice were sacrificed before operation and on the 1^st^, 3^rd^, 5^th^ and 7^th^ day after operation. The blood and terminal ileum were simultaneously collected under the aseptic condition for further usage. All procedures were performed under anesthesia with 1.5% isoflurane and 98.5% oxygen using a delivery and scavenging system designed by our laboratory [10].

### Liver function measurement

Blood samples were withdrawn from the inferior vena cava and centrifuged at a speed of 4,000 rpm for 10 minutes to isolate the serum which was stored at -80°C until use. Serum aminotransferase (ALT), total bilirubin (TB) and alkaline phosphatase (ALP) were measured in the Biochemical Laboratory of the Xingtai People’s Hospital.

### Measurement of diamine oxidase and endotoxin

Diamine oxidase (DAO) level in plasma was determined by enzyme-linked immunosorbent assay. Plasma endotoxin concentration was measured by kinetic turbidimetric limulus tests using a microorganism quick kinetic detection system (MB-80, Beijing Jinshanchuan Technology Limited Company, Beijing, China).

### Immunohistochemistry analysis

The immunohistochemical staining was performed on sections of five randomly selected samples of the terminal ileum per animal. Briefly, 5 μm paraffin sections were deparaffinized in xylene and rehydrated in a gradient of ethanol solutions. Antigen retrieval was carried out by microwave heating the sections for 20 min in citric acid buffer, and then cooling for 15 min at room temperature (RT). Endogenous peroxidase activity was quenched with 3% hydrogen peroxide at RT for 10 min. Nonspecific binding was blocked by incubating the sections for 30 min in the normal goat serum. The sections were incubated overnight at 4°C with the anti-TLR4 antibody (rabbit polyclonal antibody against TLR4; Abcam, Britain) at a dilution of 1:50, the anti-NF-κB p65 antibody (rabbit monoclonal antibody against NF-κB p65, Abcam, Britain) at a dilution of 1:800. Following several rinses in phosphate buffered saline, the sections were incubated with the anti-rabbit immunoglobulin G antibody tagged with horseradish peroxidase (BOSTER Biotechnology, Wuhan, China) for 30 min at 37°C. Finally, the sections were colored with diaminobenzidine at RT for 1**~**5 min, counterstained with hematoxylin for 5 min, dehydrated through gradient ethanol, cleared in xylene, and then mounted with permount. Images were obtained using a light microscope (CMS800; Olympus, Tokyo Japan).

### Western blot analysis

The frozen intestinal tissues were washed and homogenized on ice with RIPA buffer (Sigma-Aldrich), and centrifuged at 13000 rpm for 15 minutes at 4°C. The protein concentration in the supernatant was determined according to Bradford’s method. Proteins were resolved by sodium dodecyl sulphate polyacrylamide gel electrophoresis and transferred to a polyvinyl difluoride membrane. The blots were probed with primary antibodies against TLR4 (Abcam), NF-κB p65 (Abcam), cleaved caspase-3 (Abcam), and GAPDH (Cell Signaling Technology), followed by a secondary antibody, and their reaction products were quantified by Quantity One software (Bio-Rad Laboratories, Inc., Berkeley, CA). The relative protein expression was identified as the ratio of gray value of TLR4, NF-κB p65 and cleaved caspase-3 bands to that of GAPDH band.

### Realtime-PCR analysis

Quantitative analysis of TLR4, TRAF6, TNF-α and IL-6 mRNA was performed by Realtime-PCR. Total RNA was extracted by TRIzol reagent from Sigma. Purity and concentration of RNAs were determined by a NaNO2000 UV spectrophotometer and *OD*_260_/*OD*_280_ was between 1.8~2.2, which indicated the high purity of RNAs. According to the manufacturer’s instructions, 1μg RNA was primed with oligo (dT) using a reverse transcriptase kit from Promega. The cDNA production was amplified in an qPCR instrument from Roche. The conditions for amplification were as follows: pre-denaturation for 30s at 95°C for 1 cycle; denaturation for 10s at 95°C, annealing for 30s at 58°C and extension for 30s at 72°C for a total of 40 cycles of PCR. Three replicates were prepared for each sample in PCR detection and data were analyzed by the 2^-ΔΔCt^ method, where ΔCt = Ct _target gene_-Ct _reference gene_, and ΔΔCt = ΔCt _test group_ -ΔCt _control group_.

### Statistical analysis

The data was expressed as means ± standard deviation (SD). Quantitative variables among groups were compared with one-way ANOVA, *post-hoc* test. The correlation analysis between indicators was conducted using the Pearson or Spearman correlation test. A *p* value < 0.05 was considered significant. All statistical analyses of the experimental data were performed with SPSS 19.0 software (Chicago, IL, United States).

## Results

### Assessment of general health

The mice in the SH group remained in good health throughout the experiment. They were lively with glossy furs, normal appetite and no narcolepsy. However, for those in the OJ group, the urine turned yellow 24hrs after BDL, and skin and hair, especially ear tips and tails, turned yellow 48hrs after BDL. Drowsiness and decreased mental state and appetite were commonly observed after BDL. OJ mice ate markedly less food and there was a slowly increasing trend of body weight. On the contrary, weight gain was more significant in SH mice compared with that in OJ mice. The segment of common bile duct proximal to ligature was found dilated at collection of specimens on the 3^rd^ day after operation in OJ mice models. Swelling and yellow staining of the liver were evident on the 5^th^ day after operation.

### Continuous deterioration of liver function in mice with OJ

**Fig 1:**
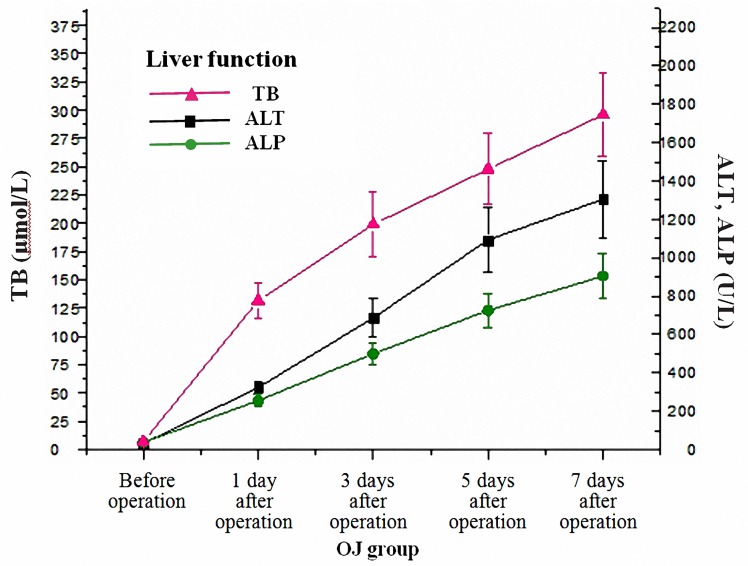
Changes of liver function in mice with time following BDL operation.

Serum levels of TB, ALT and ALP increased several hours after BDL and the liver function deteriorated with time. (Fig 1) The postoperative levels of TB, ALT, and ALP in the OJ group were compared with those before the operation and with those of the SH group at each corresponding time point, respectively, and the differences were statistically significant (*P*<0.01).

### Elevated plasma DAO level in mice with OJ

**Fig 2:**
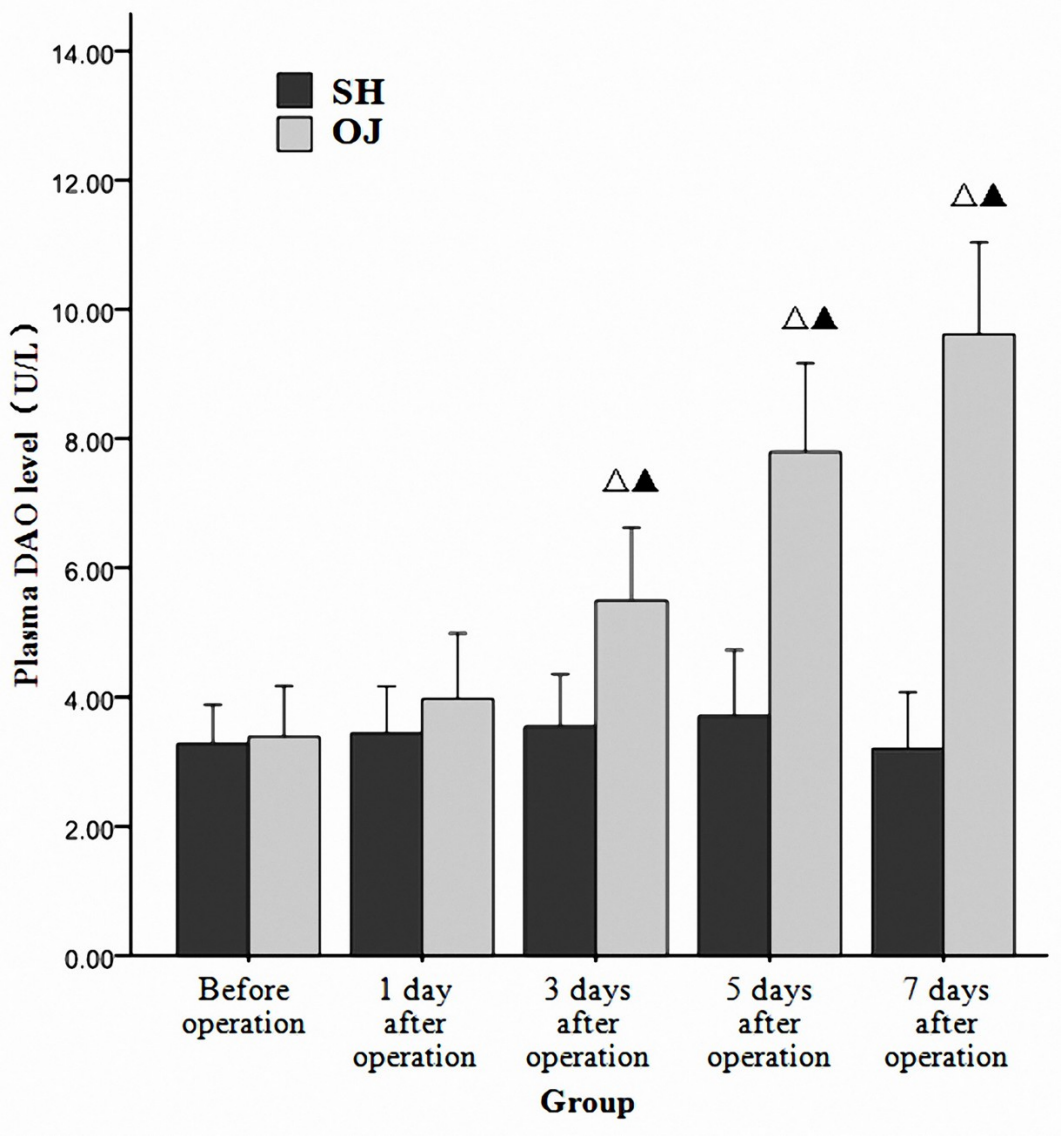
Plasma DAO level of the two groups before and after operation. ^Δ^*P* < 0.05 vs Before operation. ^*▲*^*P* < 0.05 vs SH group.

The level of plasma DAO was detected to increase progressively with time in the OJ group at different time points. Besides, plasma DAO level on the 3^rd^, 5^th^ and 7^th^ day after operation was significantly different from that before operation in the OJ group and that at corresponding time points in the SH group (*P*<0.05). However, no obvious difference was found at different time points in the SH group (*P*>0.05). (Fig 2)

### Increased plasma endotoxin level in mice with OJ

**Fig 3:**
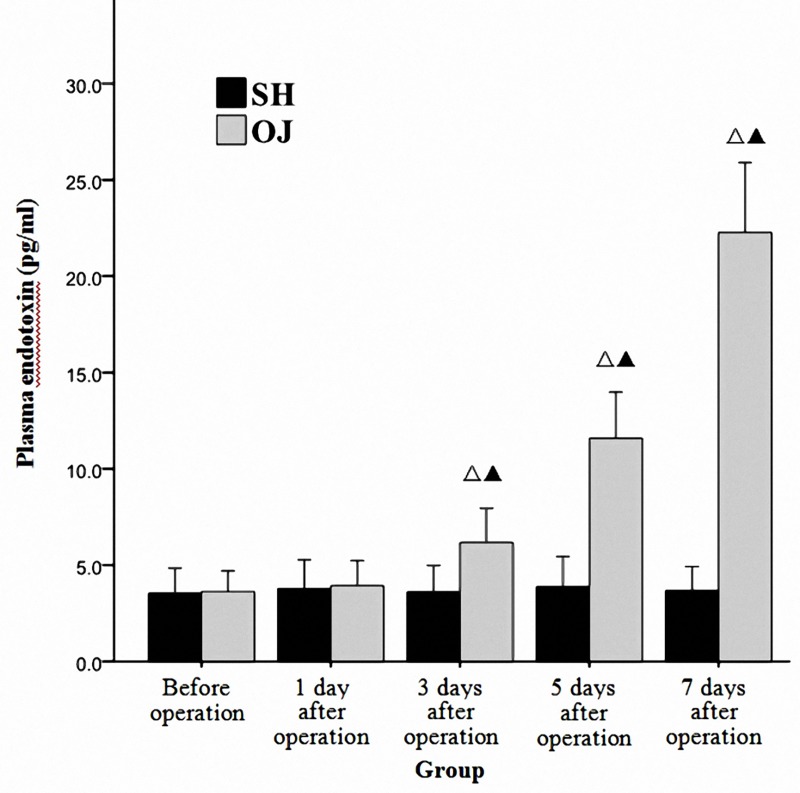
Plasma endotoxin level of the two groups before and after operation. ^Δ^*P* < 0.05 vs Before operation. ^*▲*^*P* < 0.05 vs SH group.

The level of plasma endotoxin was detected to increase progressively with time in the OJ group at different time points. Besides, plasma endotoxin level on the 3^rd^, 5^th^ and 7^th^ day after operation was significantly different from that before operation in the OJ group and that at corresponding time points in the SH group (*P*<0.05). However, no obvious difference was found at different time points in the SH group (*P*>0.05). (Fig 3)

### Increased expression of TLR4 in terminal ileum of OJ mice

**Fig 4:**
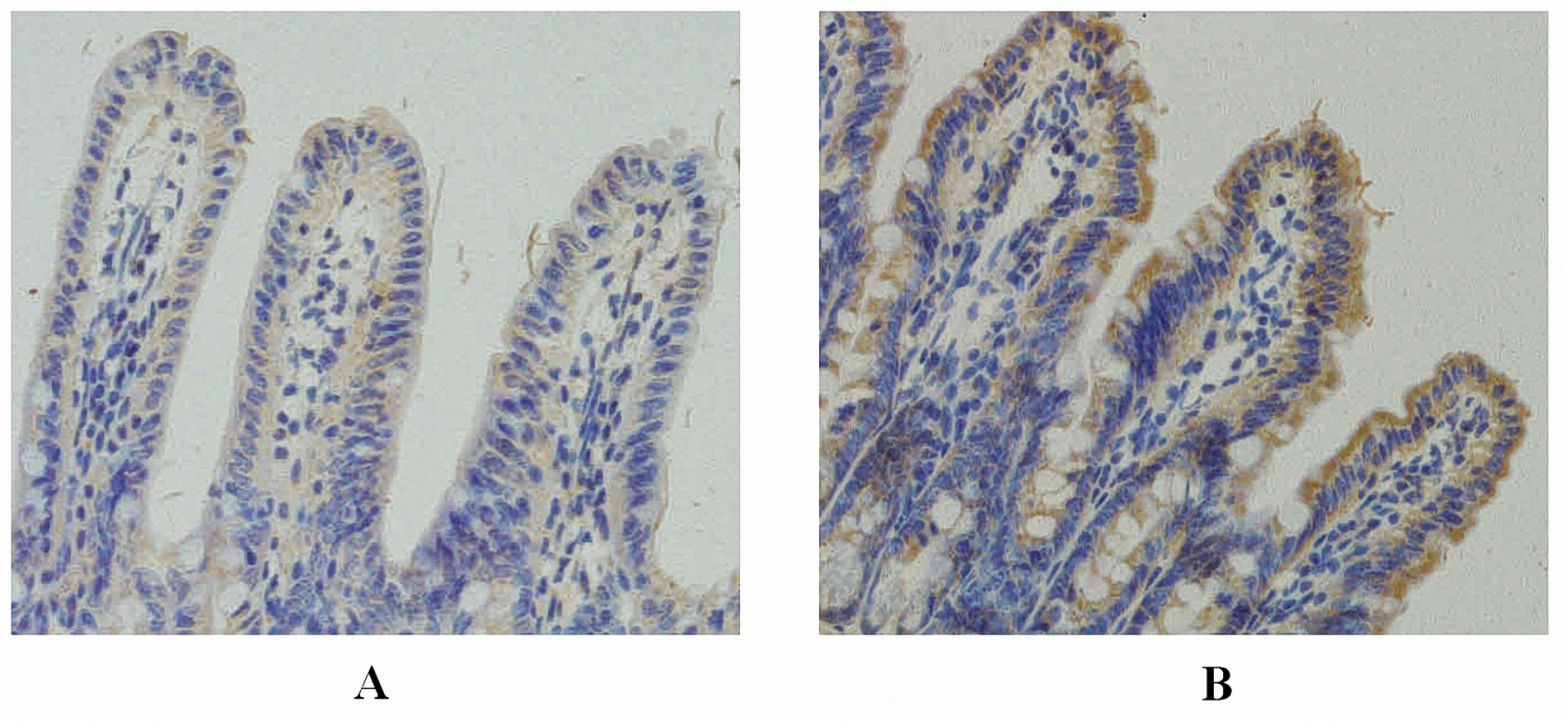
Expression of TLR4 in the terminal ileum (×200; immunohistochemistry). **A:** Immunohistochemistry revealed faint positive staining for TLR4 in the ileal epithelium of the SH group; **B:** In the OJ group, strong positive staining of TLR4 was visualized in the membrane and cytoplasm of ileum epithelial cells on the 7^th^ day after operation.

Immunohistochemistry revealed faint positive staining for TLR4 in the ileal epithelium of the SH group. In the OJ group, strong positive staining of TLR4 was visualized in the membrane and cytoplasm of ileum epithelial cells on the 7^th^ day after operation. Inflammatory cells in the interstitium also showed a few positive expressions. (Fig 4)

**Fig 5:**
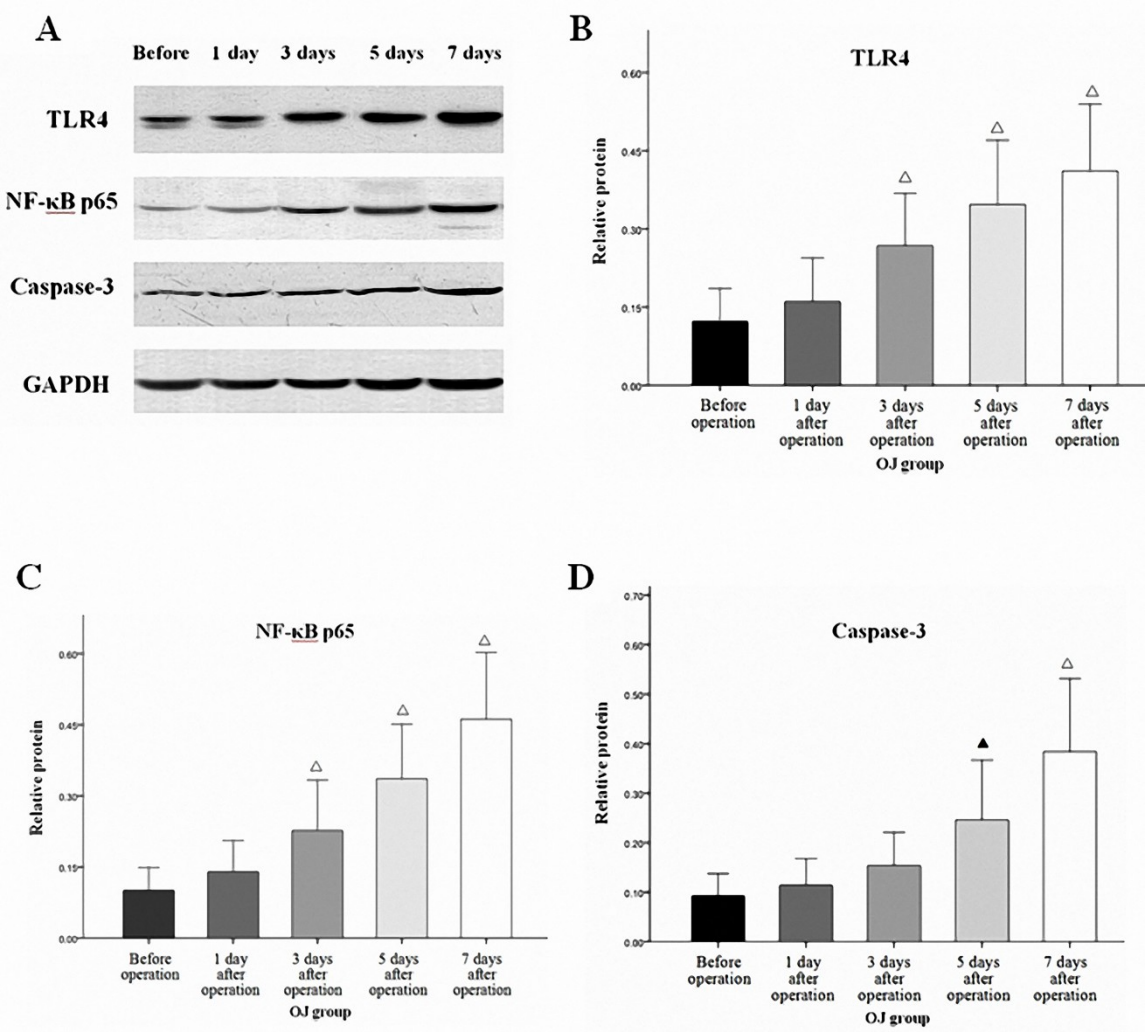
**A:** Expressions of TLR4, NF-κB p65 and cleaved caspase-3 in the terminal ileum in the OJ group (Western blot analysis). **B:** The expression of TLR4 protein increased gradually in the OJ group with the extension of time. **C:** The expression of NF-κB p65 protein increased gradually in the OJ group with the extension of time. **D:** The expression of cleaved caspase-3 protein increased gradually in the OJ group with the extension of time. ^*Δ*^*P* < 0.01 vs Before operation. ^*▲*^*P* < 0.05 vs Before operation.

There was no significant difference in the expressions of TLR4 protein and mRNA at different time points in the SH group (*p*>0.05). The expression levels of TLR4 protein in the OJ group at different time points (before operation, 1 day after operation, 3 days after operation, 5 days after operation, 7 days after operation) were 0.12±0.06, 0.16±0.08, 0.27±0.10, 0.35±0.12 and 0.41±0.13, respectively (Fig 5A and 5B). The values (2^-ΔΔCT^) of TLR4 mRNA in the OJ group at different time points were 0.49±0.19, 0.62±0.23, 0.98±0.32, 1.42±0.41 and 1.72±0.49, respectively. Both increased gradually as the obstruction time prolonged(*p*<0.05). Besides, there were obvious differences between the expressions of TLR4 protein and mRNA in the OJ group and those in the SH group on the 3^rd^, 5^th^ and 7^th^ day (*p*<0.05).

### Increased expressions of TRAF6 and NF-κB p65 in terminal ileum of OJ mice

There were no significant differences in the expressions of TRAF6 mRNA at different time points in the SH group (*p*>0.05). TRAF6 mRNA values (2^-ΔΔCT^) in the OJ group at different time points were 0.61±0.21, 0.72±0.26, 0.97±0.29, 1.28±0.37 and 1.51±0.45, respectively, which increased gradually with the extension of time (*p*<0.05). Besides, there were obvious differences between the expressions of TRAF6 mRNA in the OJ group and those in the SH group on the 3^rd^, 5^th^ and 7^th^ day (*p*<0.05).

**Fig 6:**
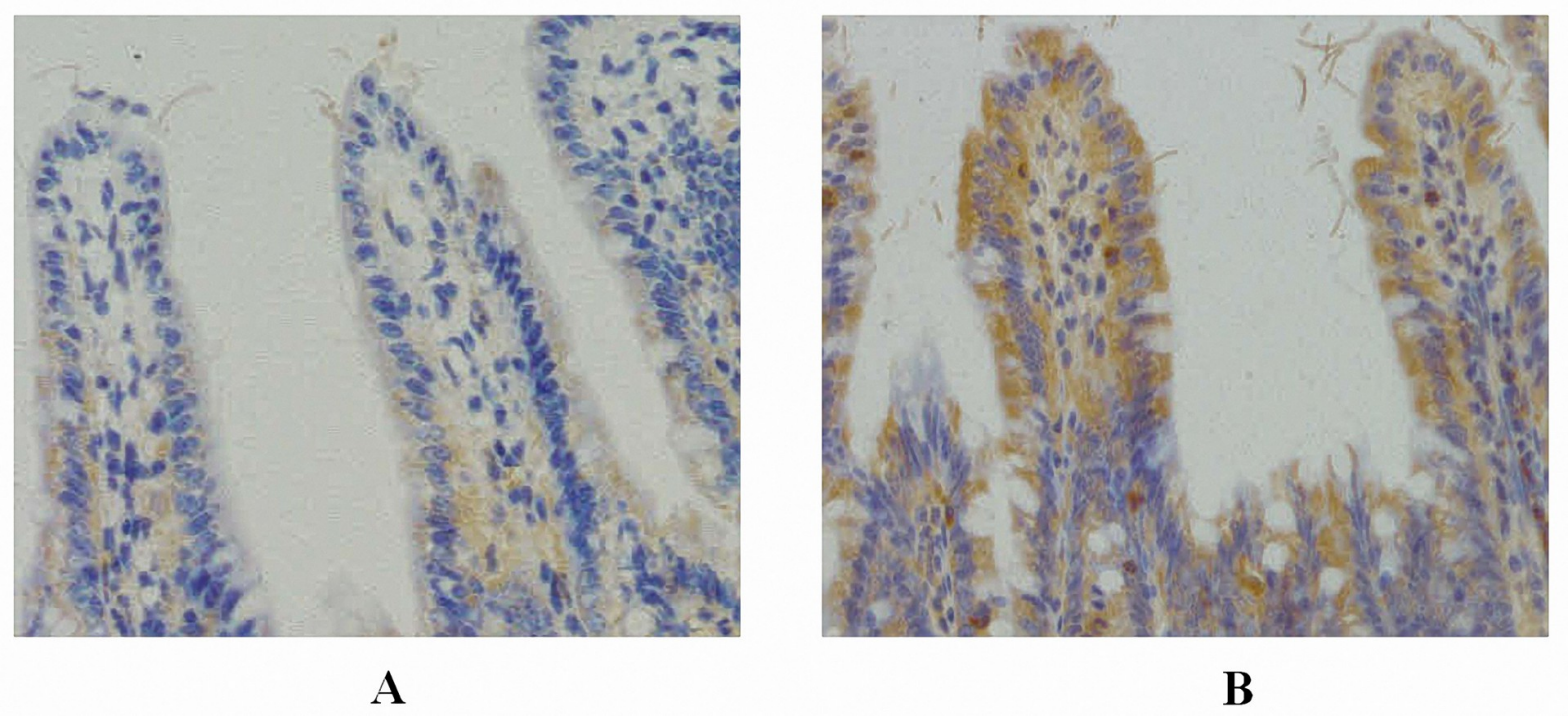
Expression of NF-κB p65 in the terminal ileum (×200; immunohistochemistry). **A:** Weakly positive expression of NF-κB p65 protein was detected in the mucosa of the terminal ileum in the SH group, and cytoplasm in a small amount of cells was light yellow with nucleus unstained; **B:** The cytoplasm in the cells of the OJ group showed enhanced brown yellow staining and visibly stained nucleus on the 7^th^ day after operation.

Weakly positive expression of NF-κB p65 protein was detected in the mucosa of the terminal ileum at different time points in the SH group, and cytoplasm in a small amount of cells was light yellow with nucleus unstained. While cytoplasm in the cells of the OJ group showed enhanced brown yellow staining and visibly stained nucleus and the staining intensity was increased over BDL time. (Fig 6) Western blot analysis showed that no significant difference in the expression of NF-κB p65 was noted at different time points in the SH group (*p*>0.05). The expression levels of NF-κB p65 protein in the OJ group at different time points were 0.10±0.05, 0.14±0.07, 0.23±0.11, 0.34±0.12 and 0.46±0.14, respectively, which increased gradually with the extension of time (*p*<0.05). Besides, there were obvious differences between the expressions of NF-κB p65 protein in the OJ group and those in the SH group on the 3^rd^, 5^th^ and 7^th^ day (*p*<0.05). (Fig 5A and 5C)

### Increased levels of TNF-α and IL-6 in mice with OJ

**Fig 7:**
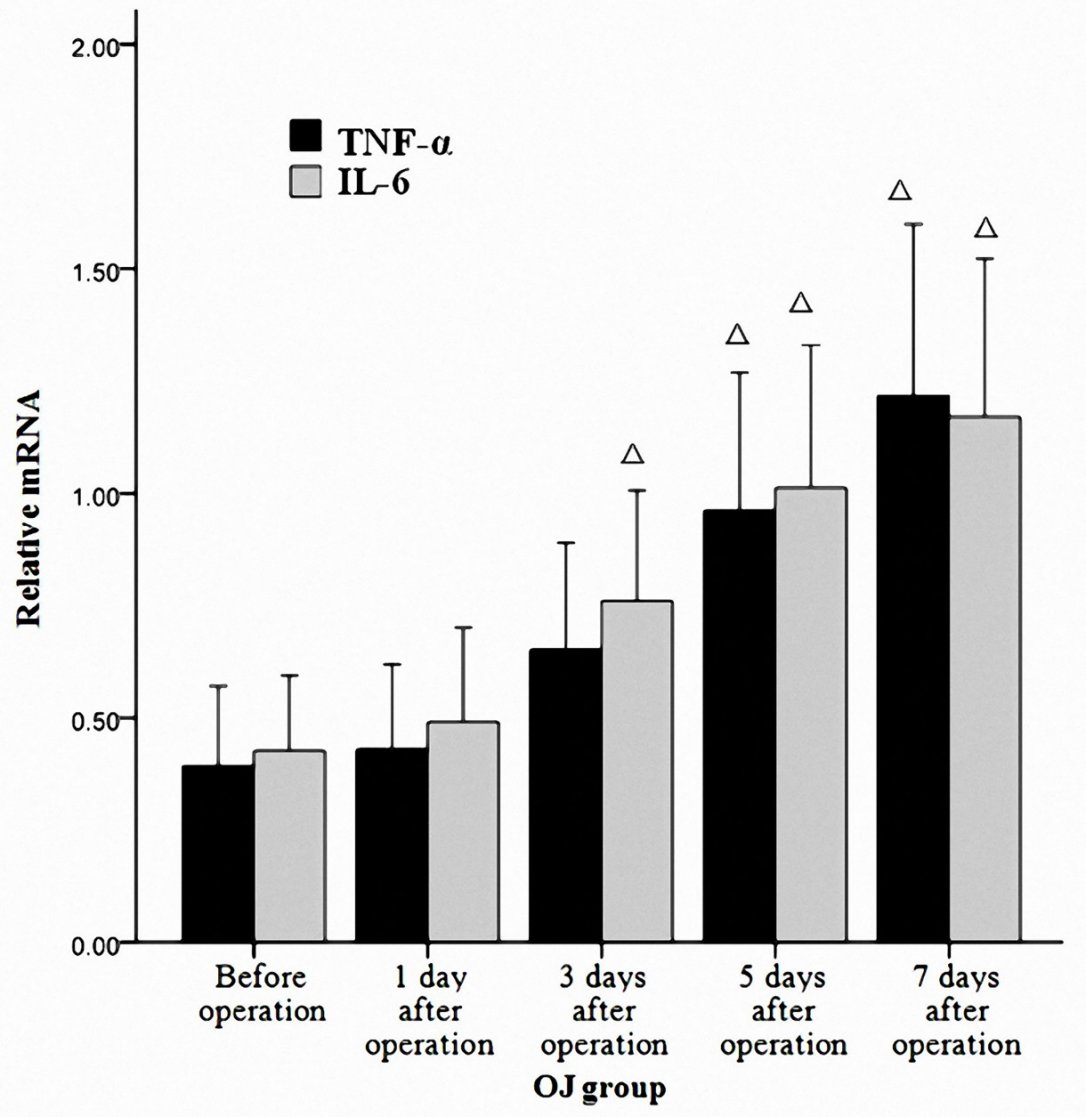
Expressions of TNF-α and IL-6 mRNA in the terminal ileum in the OJ group (2^-ΔΔCT^). The expressions of TNF-α and IL-6 mRNA increased gradually in the OJ group with the extension of time. ^*Δ*^*P* < 0.01 vs Before operation.

Realtime-PCR analysis showed that no significant difference in the expressions of TNF-α and IL-6 mRNA was noted at different time points in the SH group (*p*>0.05). However, they increased gradually as the time prolonged in the OJ group (*p*<0.05). (Fig 7) Besides, there were obvious differences between the expressions of TNF-α and IL-6 in the OJ group and those in the SH group on the 3^rd^, 5^th^ and 7^th^ day (*p*<0.05).

### Increased apoptosis of intestinal mucosa cells in mice with OJ

Western blot analysis showed that no significant difference in the expression of cleaved caspase-3 was noted at different time points in the SH group (*p*>0.05). The expression levels of cleaved caspase-3 protein in the OJ group at different time points were 0.09±0.04, 0.11±0.05, 0.15±0.07, 0.25±0.12 and 0.38±0.15, respectively, which increased gradually with the extension of time (*p*<0.05). Besides, there were obvious differences between the expressions of cleaved caspase-3 protein in the OJ group and those in the SH group on the 5^th^ and 7^th^ day (*p*<0.05). (Fig 5A and 5D)

### Correlation analysis

The expression of TLR4 had positive correlations with levels of plasma DAO (r = 0.775, *p*<0.05) and endotoxin (r = 0.691, *p*<0.05) and expressions of NF-κB p65 (r = 0.744, *p*<0.05) and cleaved caspase-3 (r = 0.706, *p*<0.05) in the OJ group.

## Discussion

OJ is a disease that can induce acute cholangitis, sepsis and multiple organ dysfunction, especially after surgery [11,12]. At present, a great quantity of studies have confirmed that OJ may impair intestinal barrier function, leads to increased intestinal permeability, as well as translocation and absorption of intestinal bacteria and endotoxin into the blood, thus leading to high risk of sepsis in OJ patients [13–15]. Therefore, the protection of intestinal mucosal impairment in the pathophysiological process of OJ and the reduction of intestinal permeability have important clinical significance in reducing the mortality rate and improving the quality of life of patients after surgery for OJ.

Common bile duct ligation in animals is a popular method used to induce OJ [16]. It was observed that serum levels of TB, ALT and ALP increased in the OJ group several hours after operation. Meanwhile, the liver function deteriorated with the prolongation of the obstruction time. Besides, obvious dilatation of common bile duct adjacent to the ligature site and obvious swelling and yellowing of the liver were also observed 3 to 5 days after operation, suggesting that the model was successfully established. DAO is a highly active intracellular enzyme in the cytoplasm of intestinal mucosal epithelial villus cells of humans and all mammals. The increase of plasma DAO suggests the destruction of the intestinal mucosal barrier and the change of intestinal permeability [17,18]. In the study, the results showed that plasma DAO level increased in the early stage of OJ and gradually with the extension of OJ time. In addition, the apoptosis of terminal ileum was analyzed by assaying cleaved caspase-3 protein. Caspase-3, a member of the cysteine-aspartic acid protease family, can be activated in the apoptotic cell both by extrinsic and intrinsic pathway [19]. Cleaved caspase-3 is an active fragment generated by the cleavage of caspase-3 during its activation. The expression of cleaved caspase-3 can thus reflect the activity of caspase-3 and apoptosis of cells. The OJ model mice obtained a high cleaved caspase-3 protein level in terminal ileum, showing that apoptosis was induced greatly. Abundant apoptosis may destruct the epithelial tight junction structures and change the mucosal barrier homeostasis [20]. Impaired intestinal barrier function and increased intestinal permeability can stimulate the translocation and absorption of endotoxin into blood. It has also been confirmed in our study that plasma endotoxin levels gradually increased with the prolonged duration of OJ. Therefore, how to protect intestinal barrier function, reduce intestinal permeability and reduce plasma endotoxin during OJ is the key to prevent intestinal infection.

TLRs are expressed in various cells in the intestinal mucosa and mainly involved in product identification and inflammatory signal transduction in pathogenic microorganisms, serving as a bridge between innate and acquired immunity [21]. Up to now 12 TLRs have been found in humans and 11 TLRs in mice, and different TLRs can specifically recognize various PAMPs on microbes [22]. Among them, TLR4 has received more attention. It can identify many endogenous and exogenous ligands. Exogenous ligands mainly involve endotoxin, including LPS of cell wall of Gram-negative bacteria [23–25]. Studies in recent years have found that the expression of TLR4 in the colon and distal ileum epithelium of the patients with IBD is significantly higher than that of normal people, indicating that TLR4 and its signal transduction pathway may play an important role in intestinal diseases. In the present study, it was found that TLR4 was slightly or weakly expressed on the mucosal surface of the terminal ileum in normal mice, which may be closely related to the immune tolerance of the intestines. Normal intestinal mucosa can tolerate the sustained stimulation from TLR ligands such as LPS and poorly respond to the external environment to maintain basic level of activation [26–32]. This is because the low level of TLR expression reduces its contact and recognition probability to PAMPs such as intestinal commensal bacteria, pathogenic bacteria or other food antigens to produce appropriate immune response and maintain the intestinal mucosal balance, namely, the intestinal mucosa homeostasis. Meanwhile, the precise negative regulation mechanism in the intestinal mucosa (extracellular, transmembrane and intracellular negative regulators) weakens the activation of TLRs and avoids the excessive intestinal mucosal immune response induced by TLRs. In the case of OJ, whether the intestinal TLR4 is activated to participate in the destruction of the intestinal mucosal barrier has not yet been reported. Inagaki et al. [33] have found that BDL can destroy the intestinal mucosal barrier and affect the expression of iNOS, IL-18 and other genes in the intestinal mucosa through relevant studies. However, the specific mechanism remains unclear, especially that the expression of TLR4 as an important upstream gene was not detected. In this study, according to Western blot and PCR, expressions of TLR4 protein and mRNA in intestinal mucosa were significantly higher in mice after common BDL than those in the SH group. Meanwhile, the expression intensity was positively correlated with the level of plasma DAO and endotoxin. Immunohistochemical results showed that increased TLR4 expression in the OJ group was mainly localized in the intestinal epithelial cell membrane. It was speculated that overexpression of TLR4 was involved in the occurrence and development of intestinal mucosal injury and endotoxemia in mice with OJ. In previous studies, we have found a significant increase in the content of intestinal gram-negative bacteria such as Escherichia coli in OJ (such data are not shown in this manuscript). TLR4 can recognize LPS of gram-negative bacteria, which is one of the main ways for the host to recognize gram-negative bacterial infection. Therefore, the increase and invasion of intestinal gram-negative bacteria may be the main cause for TLR4 overexpression. In addition, a study has confirmed that TLR4-mediated NF-κB activation in the intestine of newborns can lead to increased enterocyte apoptosis [34]. Similar results were obtained in this study. TLR4 expression was positively correlated with caspase-3 expression, suggesting that TLR4 may be involved in the apoptosis of intestinal epithelial cells in OJ. Tumor necrosis factor receptor-associated factor 6 (TRAF6), known as a key adaptor protein downstream of TLR4, plays an important role in the immune response, apoptosis, stress reaction, and inflammation [35]. This study showed that the expression of TRAF6 in intestinal mucosa increased gradually after BDL, further indicating that the activation of TLR4/TRAF6 signaling axis in OJ may be involved in cell apoptosis and injury.

NF-κB was a nuclear transcription factor widely spread in eukaryotic cells that was closely related to inflammatory responses [36]. In the resting state, NF-κB, combined with IκB, existed in the cytoplasm in an inactive form without transcription function. When the body was damaged, however, it may cause IκB phosphorylation and proteolysis and thus activate NF-κB freed from the complex [37]. The main form of NF-κB is dimer P65/P50 with a transcription activity binding site only in the C- terminal of p65, so the amount of activated NF-κB can be determined by directly measuring NF-κB p65 [38]. After having been activated, NF-κB p65 enters the nucleus and is bound to the promoter of the target gene or the κB domain of the enhancer, thereby inducing the transcription and translation of inflammatory cytokines [39,40]. The Immunohistochemical result of this study showed that NF-κB p65 was expressed in both cytoplasm and nucleus with stronger expression in the nuclei vs. the cytoplasm sometimes, suggesting that NF-κB was activated during OJ. The expressions of most inflammatory mediators, such as TNF-α and IL-6, are regulated by NF-κB during the inflammatory response [41]. TNF-α is an essential monokine in the downstream of NF-κB, which participates in the occurrence and development of most inflammation in the body. TNF-α can induce the expression of apoptosis related protein caspase-1 in epithelial cells and inhibit Bcl-2 simultaneously, thus promoting cell apoptosis [42]. IL-6 is another critical inflammatory mediator induced by the activation of NF-κB, which plays an important role in inflammatory response of local and distant organs after intestinal ischemia injury. For example, Takuya et al. [43] demonstrated that IL-6 could increase intestinal permeability by affecting the expression of claudin-2, a channel protein that constitutes the tight junction complex. In addition, TNF-α and IL-6 are also activators of NF-κB, which can form positive feedback regulation [44,45]. Under normal circumstances, a certain amount of NF-κB plays a crucial role in maintaining intestinal mucosal integrity and coordinating the innate immunity and adaptive immunity in antimicrobial activities. However, continuously high expression of NF-κB may lead to excessive release of inflammatory factors and severe inflammatory response, which aggravates the intestinal mucosal barrier destruction and starts up a vicious circle [46–48]. This study confirmed that with the extension of OJ time, the expression of NF-κB p65 was increased continuously and positively correlated with the expression of TLR4, and the levels of TNF-α and IL-6 in the intestinal mucosa gradually increased. Therefore, we speculate that the epithelial apoptosis and intestinal mucosal injury during OJ might be strongly correlated with the excessive activation of TLR4/TRAF6/NF-κB signaling pathways and the massive release of inflammatory factors.

In conclusion, the TLR4 in the terminal ileum of mice with OJ was highly expressed and activated, and massive release of inflammatory factors was induced through the TLR4/TRAF6/NF-κB signaling pathways, which was involved in intestinal mucosal injury and endotoxemia. Although the specific molecular mechanisms involved in this process require further study, we believe that inhibiting TLR4/TRAF6/NF-κB signaling will provide effective targets for the prevention and treatment of enterogenic infection during OJ.

## Abbreviations

BDL: bile duct ligation
OJ: obstructive jaundice
SH: sham operation
HE: haematoxylin-eosin
ALT: aminotransferase
ALP: alkaline phosphatase
TB: total bilirubin
DAO: diamine oxidase
TLRs: Toll-like receptors
MLNs: mesenteric lymph nodes
MAMPs: microbial-associated molecular patterns
LSD: least significant difference
IBD: inflammatory bowel disease
