# Correction: Intestinal mucosal injury induced by obstructive jaundice is associated with activation of TLR4/TRAF6/NF-κB pathways

**DOI:** 10.1371/journal.pone.0227310

**Published:** 2019-12-26

**Authors:** Xiaopeng Tian, Huimin Zhao, Zixuan Zhang, Zengcai Guo, Wen Li

In the Animal models subsection of the Materials and methods section, there is an error in the second and third sentences of the first paragraph. The correct second and third sentences are: All mice were purchased from the Laboratory Animal Center of Academy of Military Medical Sciences and housed in the Experimental Animal Service Center of the Chinese PLA General Hospital (PLAGH). The study was approved by the Animal Research Ethics Committee of the PLAGH.

In the Liver function measurement subsection of the Materials and methods section, there is an error in the second sentence of the first paragraph. The correct second sentence is: Serum aminotransferase (ALT), total bilirubin (TB) and alkaline phosphatase (ALP) were measured in the Biochemical Laboratory of the PLAGH.

In the Acknowledgements section there is an error. The correct Acknowledgements section is: The authors extend their gratitude to members of Chinese PLA General Hospital Animal Laboratory for their support during experimentation.

In the Author Contributions, Xiaopeng Tian (XT) should not be listed as contributing to funding acquisition.
